# Required Capabilities for Employee-Driven Innovation to Emerge in Healthcare Organizations: Comment on "Employee-Driven Innovation in Health Organizations: Insights from a Scoping Review"

**DOI:** 10.34172/ijhpm.8744

**Published:** 2024-11-10

**Authors:** Zakaria Belrhiti

**Affiliations:** ^1^Department of Public Health and Management, Mohammed VI International School of Public Health, Mohammed VI University of Sciences and Health, Casablanca, Morocco.; ^2^Mohammed VI Centre for Research and Innovation (CM6RI), Rabat, Morocco.

**Keywords:** Employee-Driven Innovation, Capabilities, Healthcare, CMO Configurations, Leadership, Realist Evaluation

## Abstract

Employee-driven innovation (EDI) is still under-researched in health policy and system research, particularly in low-and middle-income countries. EDI is recognized as a lever to improve the commitment of health workers, promote quality of care, and contribute to creating value and transforming healthcare practices, services, structures, and processes. The mechanisms underlying the emergence of EDI processes and outcomes include core capabilities to cope with complexity, building spaces for learning, fostering sense-making and sense-giving, and collective problem-solving. The development of such capabilities depends on organizational and individual conditions. Organizational capabilities include complex leadership, trust management practices, task complexity, and the availability of slack resources. Individual capabilities comprise capabilities to cope with complexity, such as sense-making, autonomy, system thinking, and adaptive learning. The sustainability of EDI depends on local ownership and frontline employees’ involvement during problem definition, innovation design, and implementation.

## Background

 A recent review by Cadeddu et al^1^ described employee-driven innovation (EDI) as a heterogeneous complex social process involving interwoven components: (1) innovation processes, (2) learning, (3) health workers’ engagement, and (4) digital components. However, authors have not explored how these processes led to individual outcomes (Job satisfaction, empowerment, and patient satisfaction), team-level outcomes (Improved collaboration and collective problem-solving), and organizational outcomes (Efficiency, productivity, practice improvement, quality of care, cost savings, changes in institutional and managerial practices, and sustainability).^1^ Little evidence was drawn on how to institutionalize and embed EDI in routine practice in healthcare organizations.^2,3^

 To this end, we aim to expand on the result of this review to provide plausible explanatory accounts. We have carried out a narrative review focused on EDI and innovation organizational theories. We also reflected on theoretical insights from previous review findings carried out by the first author on collaborative governance dynamics,^4^ complexity leadership,^5^ behavioral theories.^6^ We attempted to unearth potential mechanisms by using qualitative system dynamics visualization using a causal loop diagram using Vensim software to summarize and present the underlying relationship between these conditions (enablers and facilitators) and the EDI processes and outcomes. In practice, we aim to understand how EDI initiatives work (or not), why, how, and under what conditions.

**Figure 1:**
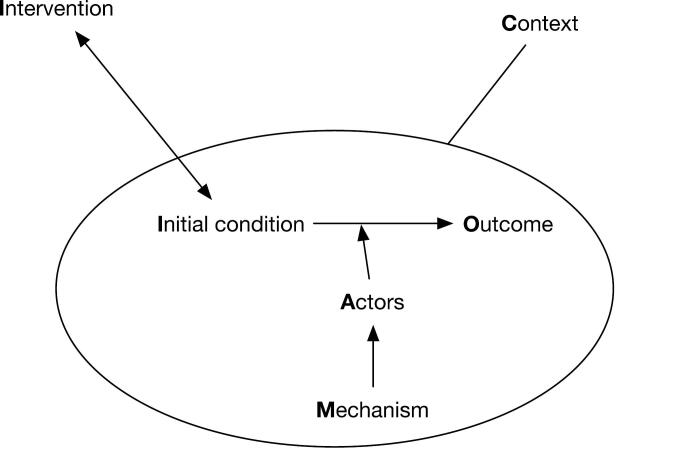
Context-Mechanism-Outcome Configuration.

 Many scholars considered EDI as complex social systems characterized by path dependency, interdependency, unpredictability, emergent processes of change.^1,7,8^ This is why realist inquiry philosophy and evidence synthesis might prove appropriate to unearth these causal pathways. The realist philosophy add insights into the identification of generative mechanisms that operate (or not) in given contextual conditions.^9^ Generative mechanisms defined as are accounts of the behavior and interrelationships of the processes which are responsible for the change and the observed regularities in specific conditions.^10^ In practice, this commentary will suggest potential causal configurations (See Figure 1) summarized in the form of Context-Mechanism-Outcome (CMO) configurations.^11^

 We refer to context as specific conditions that are not part of the intervention (physical, social, cultural, economic, and organizational environment) that enable (or hinder) the mechanisms underlying the emergence of EDI. *Mechanisms *are defined as the underlying generative forces often hidden and context-specific, including structure and agency, resources, and individual reasoning of actors and responses to their contexts.^10^ We will synthesize these CMO configurations using an initial program theory (detailed hypothesis).

 The realist perspective considers the EDI as social systems, characterized by the interactions between individuals and institutions, agency and structure, as well as micro and macro social processes as stated by Pawson and Tilley,^10^ the true causal influence resides not in specific objects or individuals, but in the social relations and organizational structures they create. Mechanisms have different forms: power and liabilities (eg, capability of individuals to learns), as forces (peer pressure), as interactions (new technology and markets innovations systems), feedback and feedforward processes (leadership, autonomy support, and high commitment), reasoning and resources (perceived organizational support and availability of resources).

 In this commentary, we refer to capabilities as a sort of power and liabilities mechanisms describing the extent to which individuals can adapt to change, generate new knowledge, and continue to improve their performance.^12^ Scholars focused on five critical individual mechanisms or capabilities to cope with complexity in EDI.^13,14^ These capabilities are derived from empirical studies of innovations in healthcare settings in low- and middle-income countries. These capabilities are organized into organizational, team and individual levels (See Box 1).

**Box 1. Core Capabilities to Foster the Emergence of Employee-Driven Innovation**

** At Organizational Level**

- Capability to engage and commit
- Capability to balance diversity and coherence (organisational levels)

** At Team Level**

- Capability to relate and attract support (Team level)

** At Individual Levels**

- Capability to carry out multiple tasks
- Capability to adapt and self-renew

Building on the evidence in the Cadeddu review and own literature review of EDI and organizational theories, we summarized key CMO in Table.

**Table:** CMOs Based on Our Review and Cadeddu et al Review

**Key CMOs**	**Definitions**
CMO 1: *Leaders’ capabilities to engage and commit*	Leaders and managers justify changes and set new agendas, influencing organizational behavior and motivation (C).^14,15^ They inspire creative problem-solving and reflective practices through sense-making practices (M). When coupled with collaborative space, it generates the emergence of profound micro-practices of *social sense-making *(M2).^a^This results in generative processes that motivate and improve management practices (C), thereby harnessing and unleashing frontline staff core capacities.This approach to problem-solving emphasizes the emergent combination of individual competencies, *collective capabilities, assets, and relationships* that enable a human system to create value. These mechanisms trigger actors' motivation and buy-in (O).
CMO 2: *Leaders’ capability to balance diversity and coherence*	Transformational leadership and Managerial (C) support also contribute to the creation of supportive *organizational culture* (C) hallmarked by psychological safety and social cohesion *trustfulness, autonomy, tolerance, and a feeling of security *(M2).* This promotes *the *perceived autonomy* of employees, buy in and ownership (O). *Balancing diversity and coherence means the ability to promotes *systemic thinking of healthcare staff, stimulates them to develop shared short- and long-term strategies and visions; balance control, flexibility, and consistency; integrate and harmonize plans and actions in complex, multi-actor settings; and cope with cycles of stability and change(C).^8^
CMO 3: *Capability to relate and attract support *	Boundary spanners (C) who can form new connections, alliances, or partnerships with others to leverage resources and actions are able to establish credibility with key stakeholders and effectively navigate competition, politics, and power dynamics.^16^ Interprofessional collaboration within healthcare organizations creates suitable conditions for appropriate knowledge flows (O), sharing of experience (O), idea generation (O), and increasing social cohesion (O). These collaborative informal spaces favor developmental and improvisational learning and creative problem-solving (M).^8^ These collaborative dynamics are catalyzed by mutual trust (M), shared understanding (M), moral engagement (M), and compatibility of values (M), a pivotal ingredient to maintaining sustainable relationships and collaboration (O)^4^ and preventing mutual suspicion, competition, and value mismatches.^17^
CMO 4: *Individual capabilities to cope with complexity *	When the organizational culture is conducive for staff participation and error trial culture (C), frontline workers are able to adapt to new task, manage multiples task while maintaining accepted levels of performance and sustaining production over time and adapt to changes (O) by engaging in self-sense making processes (M) and shared values (M), developmental and improvisational learning (M).

Abbreviation: CMO, Context-Mechanism-Outcome. ^a^ *Sense-making* involves understanding, interpreting, and creating sense by viewing problems as part of the broader dynamic system.^14,15^

 In the following paragraph, we summarized key capabilities at three different levels: organizational, team, and individual levels. This is consistent with the social multilayered social reality of realist inquiry and system thinking in health policy and system research.

## At Organizational Level

###  Leaders’ Capability to Engage and Commit 

 Leadership influence, without being in control, the creation of enabling conditions focused on the “software” of the systems (relationships, norms, knowledge, and communication) and the intersection with the “hardware” (public policy support, technology, and positional authority). Together, they serve as *sense-giving* tools by creating *shared meaning, shared understanding, moral engagement, and overcoming enforcement issues.*^14,18^ Complexity leadership literature^5^ suggest leadership needed in complex innovation context need to balance between transactional leadership, transformational and distributed leadership.

###  Leaders’ Capability to Balance Diversity and Coherence

 Transactional leadership allows to provide the coherence needs of frontline workers by clarifying goals, work group processes, and systematizing learning processes. Transactional leadership combined with management support promotes *collective actions* through *formalizing collaborative spaces* by organizing structures, providing direction and evaluation, designing job characteristics, and considering task complexity and daily challenges. They implement *external incentive policies* to reward creative work and collaborative performance.

 Transformational leadership promotes creativity by building a creative vision, forming a creative group identity, showing individual consideration, and stimulating problem-solving. Healthcare managers in complex healthcare settings must address resource constraints and structural inequalities essential for EDI. They provide necessary organizational support and ensure the availability of slack resources (finances, human resources, and time). They alleviate the tension between high commitment to innovative learning processes and other job-related demands.^19^

 Distributed leadership of frontline employees allows them to act autonomously by emphasizing quality and participation, supporting innovation and social cohesion, and promoting the formation of a *collective group identity* and a solid clan-like organizational climate and culture.^5,15^

 Distributed leadership and trust-based management fosters the *perceived autonomy* of employees, reduces micromanagement practices, and focusing on long-term outcomes is associated with EDI and Organizational citizenship behaviors in the public sector.^20^ In such complex context, managerial focus need to manage multiple relationship and distribution of power between technical, social and organizational features of innovations while being focused on middle- and long-term unknown outcomes, contributing to building a good enough vision statement and solutions.^21,22^

## At Team Levels

###  Capability to Relate and Attract Support

 Workplace learning and building collaborative spaces are a critical component of EDI as they enable frontline healthcare workers to learn through their daily tasks and social interactions.^8,23^ Indeed, interprofessional collaboration within healthcare organizations creates suitable conditions for appropriate knowledge flows, sharing of experience, idea generation, and increasing social cohesion. Workplace learning literature suggests that relationship and integration of frontline workers perspectives into social and cultural understanding of workplace leaning is critical to improving learning and are hindered by low trust relationships.^24^ Learning and innovation at the workplace requires active cognitive participation of workers referred to as “learning as participations.” Indeed, self-organization and capacity development are only possible when formal and informal social interactions and relationships are enabled within and between healthcare units and services.

## At Individual Levels

###  Capability to Adapt and Self-renew 

 The ability of health workers to self-reflect, to engage in reflective practice and adaptive learning. This ability allows them to adapt and adjust plans, engage in creative problem-solving, adapt care processes to patients’ needs, anticipate changes and new challenges proactively, learn through experience, handle changing circumstances, and build resilience.^8,23^

###  Capabilities of Frontline Workers to Carry out Multiple Tasks (eg, Technical, Clinical Logistics)

 The emergence of EDI in Healthcare organizations needs to be from a perspective of complex adaptive systems theory where multiple conditions and not a single intervention would increase health workers’ individual and collective capacities to cope with complexity, generate ideas, and develop bottom-up innovative practices, processes, and outcomes. This relies on the ability of frontline workers to generate accepted levels of performance and substantive outputs and outcomes, sustain production over time, and add value for patients and families.

**Figure 2:**
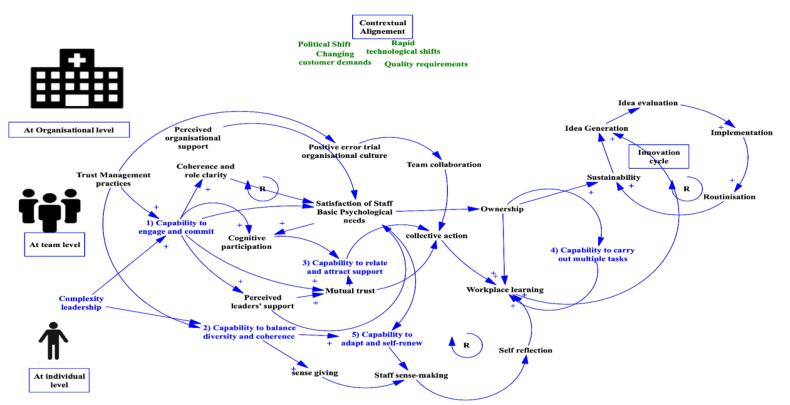
Plausible Potential Mechanisms Underlying the Emergence of Employee-Driven Innovation in Healthcare.

 We summarized the critical contextual conditions (enablers, barriers) identified in the Cadeddu et al review and selected theoretical as depicted in Figure 2. Figure 2 summarizes a causal loop diagram that identify the different required capabilities to engage in EDI and the underlying plausible causal pathways.

## Discussion

 We provided new insights into the potential plausible mechanisms underlying the emergence of EDI. The list of five capabilities reflect and validate previous realist evaluation using other implementation theories such as the normalization process theory^25^ (coherence, cognitive participation, collective action, and reflective monitoring) that aligned well with the plausible CMOs suggested in this commentary. Yet, our CMOs are propositions offering a plausible explanation that need further by empirical testing in real world settings.^10^

 Our commentary added to the literature the need to move away from inner perspective on EDI by not only integrating organizational attributes but also adopting an open innovation perspective taking into consideration the inflows and outflows of knowledge across the boundaries of healthcare organizations. Researchers might explore what are the strategies and managerial actions needed to build innovative networks, promote boundary spanning and building intra and inter organizational collaboration spaces.

 Other research might also explore the role of context, the preconditions, *how work is organized and jobs are designed and distributed, to the type of learning opportunities available to workers, the need for expansive learning environment in contrast with traditional restrictive learning environment.*^24^

 Cadeddu and colleagues did not take into consideration the role of time in sustainability of EDI. We suggest the importance of reflecting on role of Ripple Effect that is key characteristics of complex adaptive systems where the outcome of one EDI might change the context, collaborative work structures which will promotes new structures of interactions and new shared meanings^26^ (See Table, CMO 1).

 More attention needs also to be paid to the role of customization of EDI to the context, the importance of shared values, sense making and engagement and the importance of coherence between leadership practices at top level and the basic psychological needs of frontline staff. This value congruence was insufficiently addressed in the focal paper and need to be explored further in future research.

 The sustainability of EDI in healthcare organizations relies on homegrown capacity development, local actors’ ownership, and voluntary commitment, which are necessary for sustained capacities and sufficient motivation of employees to overcome resource constraints, risk-averse attitudes, coordination problems, general opposition, and stakeholder doubts.^13^

 In practice, developing, sustaining and embedding EDI in healthcare organizations requires integrated capacity development approaches focusing on both the “software” (knowledge, relationships, and norms) and “hardware” (positional authority, and policies) of the system. This is why leaders and managers must adopt a contingency mixed approach that allies software practices, including participatory methods, with hardware approaches (technology, digital tools) to ensure that EDI is negotiated and owned by all stakeholders involved rather than imposed.^14,27^

## Ethical issues

 Not applicable.

## Conflicts of interest

 Author declares that he has no conflicts of interest.
